# Screening for thyroid disease in pregnancy: a study of Danish clinical practice

**DOI:** 10.1186/s13044-023-00151-x

**Published:** 2023-03-31

**Authors:** Line Cleman Hatting, Marie Østergaard Kristensen, Maja Hjelm Lundgaard, Anne Sørensen, Stine Linding Andersen

**Affiliations:** 1 Department of Clinical Biochemistry, Aalborg University Hospital, Hobrovej 18-22, 9000 Aalborg, Denmark; 2 Department of Clinical Medicine, Aalborg University, Aalborg, Denmark; 3 Department of Gynecology and Obstetrics, Aalborg University Hospital, Aalborg, Denmark

**Keywords:** TSH, Hypothyroidism, Hyperthyroidism, Autoimmune, Gestational

## Abstract

**Background:**

Thyroid disease in pregnant women is a matter of clinical awareness, and current clinical guidelines recommend a risk-based screening strategy. This study aimed to evaluate current clinical practice regarding screening for thyroid disease in pregnancy in Denmark.

**Methods:**

A cross-sectional study was performed in the North Denmark Region with consecutive inclusion of 150 pregnant women from Aalborg University Hospital each year in 2020 and 2021. Medical records were reviewed according to the recommended risk-based screening criteria for thyroid disease in pregnancy. Any measurement of thyroid-stimulating hormone (TSH) was assessed 3 months prior to and in pregnancy.

**Results:**

Altogether 292 pregnant women who received no current treatment for thyroid disease were included. A total of 81 (27.7%) had a measurement of TSH before or during the pregnancy, and 30 women (10.3%) in the early pregnancy specifically. One or more of the screening criteria for thyroid disease recommended in the Danish clinical practice guideline were fulfilled in 37 of the 81 women (45.7%) with thyroid function tested and among 41 of the 211 (19.4%) women who did not have thyroid function tested before or during pregnancy.

**Conclusion:**

In a Danish regional investigation, 1 in 4 women had their thyroid function tested in relation to a pregnancy. However, recommended risk-based screening criteria for thyroid disease in pregnancy were heterogeneously distributed. Results encourage considerations on the current practice for the screening of thyroid function in Danish pregnant women and inform the general debate.

## Introduction

Thyroid disease in pregnant women is a matter of clinical awareness and concern. Thyroid hormones are important developmental factors, and fetal thyroid function is not fully functioning until the second half of pregnancy emphasizing the importance of maternal thyroid hormone levels in the early pregnancy [1]. Thus, much focus is on the diagnosis and management of thyroid disease in pregnancy, but uncertainties prevail regarding risk and benefits of a routine assessment of thyroid function in pregnant women [2]. Consequently, clinical guidelines recommend a risk-based screening for thyroid disease in pregnancy [3, 4].

The rationale of a risk-based screening strategy is to assess thyroid function among pregnant women who carry a higher risk of having undetected thyroid disease. This is specified in clinical practice recommendations via the listing of risk factors to be evaluated in women seeking pregnancy or newly pregnant [3, 4]. If minimum one of the listed criteria is fulfilled, measurement of thyroid-stimulating hormone (TSH) is recommended [3, 4]. The screening criteria include among others patient or family history of thyroid disease or other autoimmune diseases, reproductive history, and maternal characteristics such as age and body mass index (BMI) as well as population iodine intake. However, the set of criteria varies between international and national guidelines, as seen for the comparison of the risk-based screening criteria proposed by the American Thyroid Association (ATA) and the Danish Endocrine Society (DES) (Table 1).

**Table 1:** Risk-based screening criteria for thyroid disease in pregnant women included (x) in clinical practice guidelines

	**DES**	**ATA**
**Maternal characteristics**		
Age > 30 years		x
BMI ≥ 40 kg/m^2^	x	x
**Reproductive history**		
Multiple prior pregnancies (≥ 2)		x
Previous preterm birth	x	x
Previous pregnancy loss		x
Recurrent pregnancy loss (≥ 3)	x	
Fertility treatment	x	x
**Family history**		
Family history of thyroid disease	x	x
**Disease history**		
Use of amiodarone, lithium, or recent iodinated contrast		x
History of radiation therapy of the head and neck area	x	x
Type 1 diabetes	x	x
Other autoimmune diseases	x	x
Previous thyroid disease	x	x
Known thyroid autoantibody positivity^a^	x	x
**Disease symptoms or signs**		
Symptoms or signs of thyroid disease^a^	x	x
Presence of goiter^a^	x	x
**Demographics**		
Residing in an area of moderate-severe iodine deficiency^b^		x

^a^Not assessed in the study because information was not available in the records

^b^Not assessed in the study because this criterion would apply to all women studied

Abbreviations: *DES* Danish Endocrine Society, *ATA* American Thyroid Association

In Denmark, the recommended risk-based screening for thyroid disease in pregnant women is typically carried out at the first visit in general practice (GP) offered to every woman in pregnancy week 6–10. The Danish population was previously iodine deficient with regional differences, and a mandatory iodine fortification of salt has been implemented since the year 2000. However, iodine intake was still below recommendations when evaluated in the North Denmark Region (moderate iodine deficiency) despite an increase in population iodine levels from 2019 and onwards [5, 6]. Thus, the screening criteria recommended in Denmark do not include population iodine intake as this criterion would effectively be a universal screening [3]. Furthermore, maternal age is not currently included as a screening criteria in Denmark [3].

Uncertainties prevail regarding the choice of criteria in a risk-based screening strategy, and sparse data are available to substantiate whether the recommendations of a risk-based screening are being complied with [7]. To inform the debate, a retrospective evaluation of current clinical practice in a Danish region regarding risk-based screening for thyroid disease in pregnant women was performed, and national screening criteria (DES) as well as internal recommendations (ATA) were considered.

## Materials and methods

### Study population

**Fig. 1:**
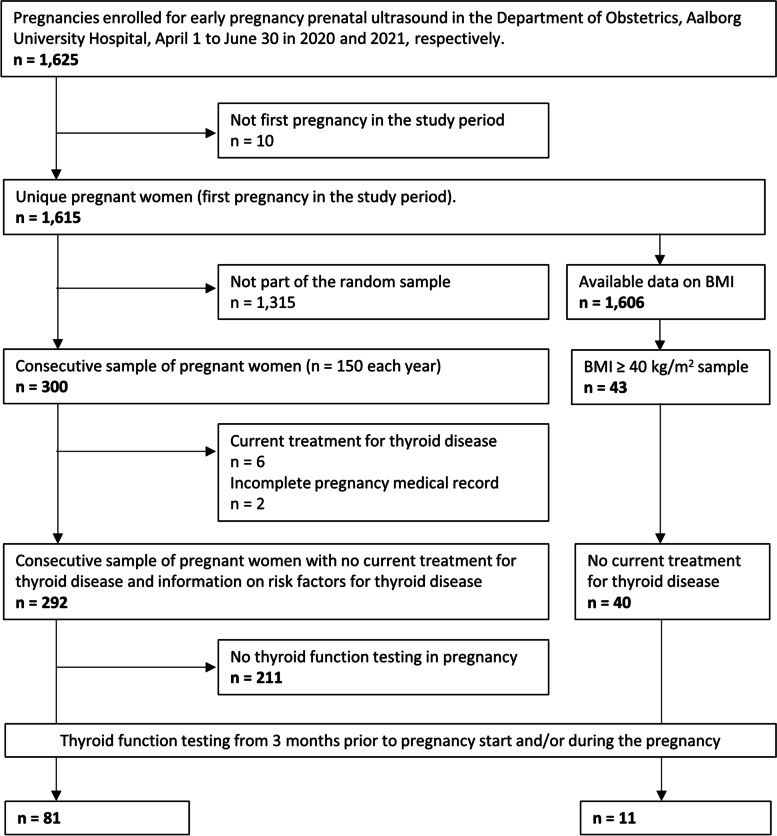
Flowchart of study inclusion

This is a cross-sectional study of pregnant women in the North Denmark Region who attended fetal ultrasound as part of prenatal screening at Aalborg University Hospital from April 1 to June 30 in 2020 and 2021, respectively (Fig. 1). In Denmark, all pregnant women are offered routine visits in GP and as part of the first trimester visit, a standard pregnancy medical record is filled out and the woman is offered participation in the national prenatal screening for chromosomal anomalies including a blood sample drawn in pregnancy week 8–10 and a fetal ultrasound, which is carried out at public hospitals in pregnancy week 11–14 [8]. All public obstetric hospital departments use a clinical fetal medicine database (Astraia Software GMBH, Germany) as part of the electronic medical record which contains information on maternal characteristics and obstetric ultrasound examinations. Study participants in the full sample were identified via Astraia, and we a priori decided on a consecutive sample of 300 pregnant women corresponding to 5% of all births annually in the North Denmark Region [9]. We included 150 pregnant women from April 1st in each of the years 2020 and 2021, because we hypothesized that the Coronavirus disease 2019 (COVID-19) shutdown, which was predominant in Denmark in the year 2020, could have influenced the management of patients.

### Data collection

Information from Astraia was assessed for the full cohort (maternal age, parity, pre-pregnancy BMI, smoking, ethnicity, mode of conception, and ultrasound-determined gestational age and date of birth). Information from the pregnancy medical record focused on assessment of the recommended risk-based screening criteria for thyroid disease (Table 1). The record has predefined questions on maternal pregnancy history including previous pregnancy loss, preterm birth, maternal chronic disorders, and use of medicine. Information on maternal chronic disorders is a tick-off box with thyroid disease and diabetes being among the disorders mentioned. Furthermore, a free text field on previous inpatient hospital visits of relevance for the pregnancy is part of the record. In addition to the standard pregnancy record from GP, the hospital medical record was assessed for any information regarding previous or current thyroid disease or other autoimmune diseases. The criteria of known thyroid autoantibody status and symptoms or signs of thyroid disease were not evaluated because this information was not specifically noted in the medical record. The evaluation was performed in the consecutive sample and among all women in the full cohort with a BMI ≥ 40 kg/m^2^ (Fig. 1). The inclusion criterion regarding BMI was chosen because it is part of the risk-based screening criteria in both guidelines (Table 1). Obesity has been associated with thyroid function in non-pregnant [10] and pregnant individuals [11], but we hypothesized that the awareness among GPs on the link between BMI and thyroid disease was poor, and we evaluated this criterion in detail.

For women in the consecutive and the BMI sample, information was available on all patient blood samples drawn as part of clinical care in the North Denmark Region Clinical Laboratory Information System II. Biochemical analyses were evaluated from 3 months prior to pregnancy start until the date of pregnancy termination. If thyroid function was tested, the TSH concentration was registered, and it was considered whether other thyroid function tests were measured or thyroid peroxidase antibodies (TPO-Ab). In the North Denmark Region, clinical laboratories use a harmonized reference interval for TSH in non-pregnant adults (0.3–4.5 mIU/L). In pregnancy, trimester-specific reference intervals for maternal TSH are recommended in Denmark (first trimester 0.1–3.5 mIU/L, second trimester 0.3–3.5 mIU/L, third trimester: 0.3–3.5 mIU/L) when local, method-specific reference intervals are not established, and were used in this study [3].

To evaluate whether COVID-19 could have affected clinical practice, 10 random GPs in the North Denmark Region were contacted by phone to provide information on their standard procedure for the management of the first pregnancy visit including the pregnancy record form. We evaluated whether a medical doctor or other health professionals filled out the form and whether the pregnant woman was attending in person.

### Statistical analyses

All data were collected and managed using Microsoft Excel (Microsoft Corporation, Redmond, USA). Results were reported as continuous variables using the median, and interquartile range (IQR) or as categorical variables using the number of cases (n) and frequency (%). Subgroups with less than three cases were reported as < 3 according to local data regulations. Statistical comparison of categorical variables was performed using the Chi-squared test. Analyses were performed using Stata 17.0 (StataCorp, Texas, USA).

## Results

In the full sample of 1,615 pregnant women, median maternal age was 29.6 years (IQR 26.9–32.9 years), 50% were nulliparous, and median BMI was 24.8 kg/m^2^ (21.9–28.7 kg/m^2^). Overall, maternal characteristics did not differ in the consecutive sample of 300 women and when stratified by the year of inclusion (Table 2). In the consecutive sample, 6 women received current treatment for thyroid disease (Levothyroxine) and were excluded from subsequent analyses as were 2 women with incomplete pregnancy medical record (Fig. 1). Among the 292 pregnant women studied, 81 (27.7%) had TSH measured in a blood sample at least once during the period from 3 months prior to pregnancy until the end of the pregnancy, and 30 women (10.3%) had a blood sample drawn in the first trimester of pregnancy specifically.

**Table 2:** Characteristics of samples (full and consecutive) and the consecutive sample stratified by year of inclusion

	**Full sample**		**Consecutive sample**		**2020**^**a**^		**2021**^**a**^	
	*n*	%	*n*	%	*n*	%	*n*	%
**Pregnant women**	**1,615**		**300**		**150**		**150**	
**Age**								
< 25 years	174	10.8	28	9.3	18	12.0	10	6.7
25–30 years	686	42.5	115	38.3	59	39.3	56	37.3
> 30 years	755	46.8	157	52.3	73	48.7	84	56.0
**Parity**								
0	818	50.7	153	51.0	72	48.0	81	54.0
1	576	35.7	108	36.0	58	38.7	50	33.3
> 2	221	13.6	39	13.0	20	13.3	19	12.7
**BMI**^b^								
< 25 kg/m^2^	830	51.7	151	50.3	71	47.3	80	53.3
25–30 kg/m^2^	459	28.6	88	29.3	49	32.7	39	26.0
> 30 kg/m^2^	317	19.7	61	20.3	30	20.0	31	20.7
**Smoking**								
Current	93	5.8	13	4.3	5	3.3	8	5.3
Previous	21	1.3	12	4.0	5	3.3	7	4.7
Never	1,501	92.9	275	91.7	140	93.3	135	90.0
**Ethnicity**^b^								
Caucasian	1,542	95.5	288	96.3	144	96.0	144	96.6
Other	72	4.5	11	3.7	6	4.0	5	3.4
**Conception**^b^								
Spontaneous	1,429	88.5	254	85.0	123	82.0	131	87.9
Other	185	11.5	45	15.0	27	18.0	18	12.1

^a^Comparison of 2020 versus 2021 within the consecutive sample using Chi-square test: *p* > 0.05 for all variables

^b^Missing data not included: BMI (*n* = 9), ethnicity (*n* < 3), conception (*n* < 3)

**Fig. 2:**
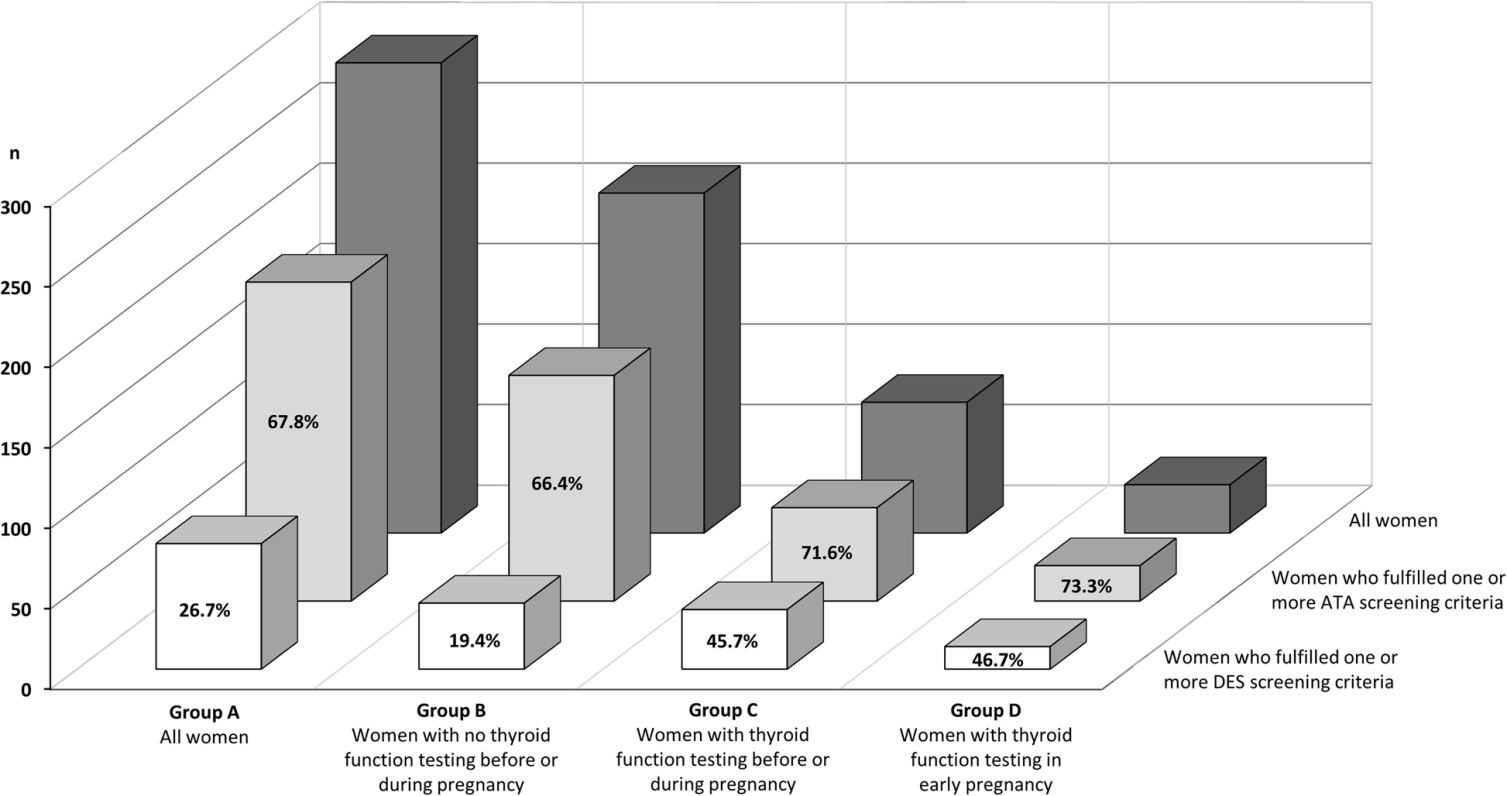
Number of women (*n*) who fulfilled minimum one or more of the risk-based screening criteria for thyroid disease in pregnancy recommended by the Danish Endocrine Society (DES) or the American Thyroid Association (ATA) when evaluated among the 292 women with no current treatment for thyroid disease and available information from the review of medical records. Results are illustrated among all women (group A) and stratified according to whether maternal thyroid function was tested before or during the pregnancy (groups B-D). Percentages are the percentages of all within each group A, B, C, and D who fulfilled one or more of the DES criteria or the ATA criteria for risk-based screening

The evaluation of the screening criteria for thyroid disease overall revealed that 78 (26.7%) of the 292 women fulfilled one or more criteria in the DES guideline, whereas 198 (67.8%) fulfilled minimum one criterion of the ATA (Fig. 2). The DES criteria were fulfilled in nearly 20% and the ATA criteria in two third of the cases when thyroid function was not tested, and in nearly 70% of the cases when maternal thyroid function was tested before or during pregnancy or in early pregnancy specifically (Fig. 2). For the DES criteria, most women fulfilled a single criterion, whereas for the ATA recommendations, a larger proportion of women fulfilled 2 or more criteria (Table 3). For individual screening criteria, maternal age > 30 years was most frequently observed with an equal distribution according to whether maternal thyroid function was tested. This was followed by fertility treatment that occurred more frequent in the group of women tested (Table 3).

**Table 3:** Risk-based screening criteria among women in the consecutive sample when stratified by thyroid function testing

	**Thyroid function tested**^**a**^		**Thyroid function not tested**^**b**^	
	*n*	%	*n*	%
**Pregnant women**^**c**^	**81**		**211**	
**Screening criteria, overall**				
**DES criteria**	**37**	**45.7**	**41**	**19.4**
1 fulfilled	31	38.3	36	17.0
2 or more fulfilled	6	7.4	5	2.4
**ATA criteria**	**58**	**71.6**	**140**	**66.4**
1 fulfilled	24	29.6	62	29.4
2 or more fulfilled	34	42.0	78	37.0
**Screening criteria, specific**				
**Maternal characteristics**				
Age > 30 years	42	51.8	111	52.6
BMI ≥ 40 kg/m^2^	< 3	-	6	2.8
**Reproductive history**				
Multiple prior pregnancies (≥ 2)	6	7.4	30	14.2
Previous preterm birth^d^	4	4.9	5	2.4
Previous pregnancy loss^d^	20	24.7	55	26.2
Recurrent pregnancy loss (≥ 3)^d^	< 3	-	< 3	-
Fertility treatment^d^	24	29.6	21	10.0
**Family history**				
Family history of thyroid disease^d^	< 3	-	6	2.9
**Disease history**				
Use of amiodarone, lithium, or recent iodinated contrast	< 3	-	< 3	-
History of radiation therapy of the head and neck area	< 3	-	< 3	-
Type 1 diabetes	6	7.4	< 3	-
Other autoimmune diseases	< 3	-	5	2.4
Previous thyroid disease^d^	3	3.8	< 3	-

Abbreviations: *DES *Danish Endocrine Society, *ATA *America Thyroid Association

^a^Measurement of maternal thyroid-stimulating hormone (TSH) from 3 months prior to pregnancy or during the pregnancy

^b^No measurement of maternal thyroid-stimulating hormone (TSH) from 3 months prior to pregnancy or during the pregnancy

^c^Women included received no current treatment for thyroid disease and had available data on risk factors for thyroid disease

^d^Missing data not included: previous preterm birth (*n* < 3), previous pregnancy loss (*n* < 3), recurrent pregnancy loss (*n* < 3), fertility treatment (*n* < 3), family history of thyroid disease (*n* < 3), previous thyroid disease (*n* = 4)

Thyroid function test results were evaluated among women tested before or in pregnancy. Considering the women’s first TSH measurement in each time window, median TSH was 1.6 mIU/L before and 1.8 mIU/L in pregnancy with samples being drawn in median pregnancy week 12. The frequency of abnormal TSH was low (TSH in the range from 3.6–4.5 mIU/L: before pregnancy: *n* < 3; in pregnancy *n* = 3 (5.5%), from 2.5–3.5 mIU/L: before pregnancy: *n* = 7 (20.6%); in pregnancy *n* = 10 (18.2%)), and TPO-Ab were rarely measured (6 of 81 women tested (7.4%)). Altogether 23.5% of women tested before and 21.8% of women tested in pregnancy had a repeated TSH measurement in the pregnancy. None of the women tested initiated treatment for thyroid disease during the pregnancy.

A total of 43 women in the full sample (2.7%) had BMI ≥ 40 kg/m^2^ (Fig. 1), and 3 women received treatment for thyroid disease leaving 40 women for evaluation with a median age of 30.7 years (IQR 28.2–33.6 years) and 32.5% expecting their first child. In total 11 of 40 women (27.5%) had TSH measured before and/or in pregnancy and maternal TSH was median 1.7 mIU/L before (*n* = 7) and 1.4 mIU/L in pregnancy (*n* = 8).

Frequencies of thyroid function testing did not differ when stratified by year of inclusion (before or during the pregnancy: 27.7% in 2020 and 27.4% in 2021 (*p* = 0.95), in early pregnancy: 8.8% in 2020 and 11.6% in 2021 (*p* = 0.42)). Evaluation among 10 random GPs showed that screening for thyroid disease was managed by a doctor in 20% of the GPs and by other professionals (nurse, midwife, laboratory assistant, or secretary) in 80% of the cases. In all GPs the visit was carried out in person, however, in the year 2020 half of the GPs performed the first visit via telephone.

## Discussion

### Principle findings

This regional study provides insight into clinical practice for thyroid function testing in Danish pregnant women. Results of this retrospective evaluation highlight that 25% of pregnant women in the North Denmark Region had their thyroid function tested before or during pregnancy. Less than half of the women screened fulfilled one or more of the risk-based criteria in the national guideline, whereas one or more criteria were fulfilled among 20% of women not screened. These proportions increased markedly when the additional criteria for risk-based screening recommended in an international guideline (maternal age, parity, and complete history of pregnancy loss) were included. Results encourage considerations on the current clinical practice regarding risk-based screening for thyroid disease in Danish pregnant women and inform the general debate.

### Interpretation

The important role of maternal thyroid function in pregnancy has long been recognized [2]. The hypothesis that abnormal maternal thyroid function when left untreated may adversely affect outcomes of a pregnancy and fetal development is biologically plausible [12]. Furthermore, retrospective evaluation of blood samples from large cohorts across populations have shown that thyroid function abnormalities occur in pregnant women compatible with the fact that symptoms of thyroid dysfunction are often unspecific, and that thyroid disease may exist unidentified [13]. An established test for assessment of maternal thyroid function is available, but inadequacies prevail regarding the implementation of population-, method- and trimester-specific reference intervals, and unresolved matters regarding screening for thyroid disease in pregnant women point to the definition of abnormal thyroid function and the indication for and outcome of treatment [14].

Benefits and risks of screening for thyroid disease in pregnant women remains controversial including the choice of screening strategy [4]. Current guidelines do not advocate routine testing of thyroid function in all pregnant women, but recommend a risk-based (selective) screening aiming to identify women with the highest risk of suffering from undetected thyroid disease [3, 4]. A series of studies compared universal and selective screening strategies and the overall study conclusions point to the fact that a substantial number of women with abnormal thyroid function are missed with a selective screening as compared to universal screening [15]. The aim of the present study was not to compare risk-based screening against other screening strategies or to evaluate outcome of pregnancy but aimed to evaluate whether current clinical practice for risk-based screening is being complied with in Denmark. This is the first report on this matter in Denmark, and sparse data are similarly available from other countries. In 2004, Haddow et al. surveyed 61 prenatal care practices in Maine and found an overall screening rate of 48%, however, the rate differed by responsible physician and was 56% among obstetricians and 8% among family doctors [16]. In 2008, Chang et al. studied 983 pregnant women attending Boston Medical Center and found screening rates of 84% and 86% among obstetricians and family doctors [17]. In 2010, the European Thyroid Association (ETA) conducted a survey among their members (mostly endocrinologists with GPs accounting for 2%) and found that 42% of responders would perform universal screening, 43% selective screening, and 17% no screening of pregnant women [7]. Thus, reports of screening rate and practice vary considerably, and study comparison is difficult since health care systems and the management of pregnant women may also vary across populations.

In Denmark, all pregnant women are offered pregnancy care visits in GP, and thereby this study evaluates the screening rate among family doctors in Denmark. Our report raises a concern about the current strategy for risk-based screening in Denmark considering the heterogenous distribution of risk factors according to whether thyroid function was tested. We speculate on the knowledge among GPs regarding the recommended screening strategy. Our survey in a small subset of GPs substantiates that other health care professionals may fill out the pregnancy medical record form including the information on thyroid screening and that the information may in some cases be obtained via telephone. Less than half of the women screened in our study had risk factors for thyroid disease. Even if information on any ongoing symptoms or signs of thyroid disease or previous elevated thyroid autoantibodies was not available, we find it unlikely that these factors would be the indication for thyroid function testing in more than half of the women. Considering the frequency of the different risk factors in our study population, we found a low frequency of for example family history of thyroid disease compared with other reports [18, 19], thus, we speculate on the feasibility of the uniform medical record used during the first pregnancy visit in GP in Denmark which is not designed with specific thyroid questions except for the tick box to mark maternal chronic diseases including thyroid disease. Information on maternal socioeconomic status, including educational level, is not registered in the pregnancy record, thus, we were unable to evaluate the role these factors in relation to current screening practice.

We observed different proportions of risk factors according to whether the Danish or the international guideline was considered, and a change in the Danish guideline to comply with the ATA recommended screening criteria would more than double the number of Danish women fulfilling a criterion for being screened. In our study population, 60–70% of the women would fulfilled an ATA criterion, which is in line with a report from Belgium (64%) [20] and higher than in Iranian pregnant women (44%) [19]. In all populations, the age criterion appears predominant, particularly when average maternal age is approximating 30 years which is the case in for example Denmark and Belgium. In addition to age, we specifically evaluated all women in the full cohort with BMI ≥ 40 kg/m^2^ since this risk factor is part of the Danish and the ATA guideline. We found that 2.7% of the women classified with morbid obesity in our study population with corresponding figures of 0.5% in Iran [19] and 1.3% in Belgium [20]. Only 25–30% of these women had their thyroid function tested in our population, and we speculate on the awareness among GPs on this specific criterion. In line with this consideration, less than 20% of responders in the 2016 ETA survey would carry out screening of pregnant women according to this specific risk factor [7]. Maternal age and BMI are examples of risk factors that may vary between populations and over time within a population. Similarly, the screening criteria included in the ATA guideline [4] on moderate-severe iodine deficiency is population-specific and a change in population iodine intake, for example with iodine fortification, may influence the screening practice according to this criterion. The present study did not allow for evaluation of this criterion, because it is not included in the Danish guideline [3], and because it would imply universal screening in the region studied according to the most recent evaluation of iodine intake in pregnant women within this region [6].

### Methodological comments

We studied a consecutive cohort of pregnant women which was comparable to the full cohort considering maternal characteristics. The women were identified because obstetric ultrasound was carried out as part of prenatal screening for chromosomal anomalies. The rate of participation is high [8], however, women with early pregnancy loss prior to prenatal screening were not identified. We studied 300 pregnant women in the consecutive sample which was the protocolled number according to the study aim and data availability. It was not a primary study endpoint to estimate the prevalence of abnormal thyroid function in pregnancy and the sample size should be acknowledge considering these results, e.g., the prevalence of unknown overt hypothyroidism is 0.2–0.3% in pregnant women, and the low frequencies of abnormal thyroid function reported are to be expected [2]. We a priori decided on the time window for assessment of biochemical test results in relation to the pregnancy under study, however, misclassification cannot be excluded regarding the assessment of thyroid function testing in relation to ongoing fertility treatment and information on such treatment in private hospitals. Our study was regional and confined to a single hospital in the North Denmark Region. The Danish clinical guideline [6] is valid and recommended for the entire country; however, clinical practice and maternal characteristics may differ across regions, and our findings may not apply to other populations.

## Conclusion

In a Danish regional investigation, the adherence to national and international recommendations on risk-based screening for thyroid disease in pregnancy was evaluated. Overall, 25% of women had their thyroid function tested before or during the pregnancy, but the distribution of risk factors for thyroid disease was heterogenous. Thus, the national recommended screening criteria were fulfilled in less than half of the women screened, and in 1 of 5 women not screened. Results raise a concern about compliance with the current screening recommendations, inform the debate on screening for thyroid disease in pregnant women, and encourage further evaluation in Denmark and across populations.

## Data Availability

The data generated and analyzed during the study are not publicly available to ensure individual confidentiality but can be made available from the corresponding author upon reasonable request.
